# Clinical Impact of Spontaneous Hyperactivity in Degenerating Retinas: Significance for Diagnosis, Symptoms, and Treatment

**DOI:** 10.3389/fncel.2018.00298

**Published:** 2018-09-10

**Authors:** Steven F. Stasheff

**Affiliations:** ^1^Center for Neuroscience and Behavioral Medicine, Gilbert Family Neurofibromatosis Institute, Children’s National Health System, Washington, DC, United States; ^2^Visual Neurophysiology, Neuro-ophthalmology and Pediatric Neurology, Retinal Neurophysiology Section, National Eye Institute, Bethesda, MD, United States

**Keywords:** retinal degeneration, hyperactivity, retinal ganglion cell (RGC), clinical impact, retinal remodeling, visual perception, neural encoding/decoding, visual restoration

## Abstract

Hereditary retinal degenerations result from varied pathophysiologic mechanisms, all ultimately characterized by photoreceptor dysfunction and death. Hence, much research on these diseases has concentrated on the outer retina. Over the past decade or so increasing attention has focused on concomitant changes in complex inner retinal neural circuits that process visual signals for transmission to the brain. One striking abnormality develops before the ultimately profound anatomic disruption of the inner retina. Highly elevated spontaneous activity was first demonstrated in central nervous system visual centers *in vivo* by Dräger and Hubel (1978), and subsequently has been confirmed *in vitro*, now in multiple animal models and by multiple investigators (see other contributions to this Research Topic). What evidence exists that this phenomenon occurs in human patients with retinal degeneration, and what is the ultimate effect of spontaneous hyperactivity in the output neurons, the retinal ganglion cells? Here I summarize abnormalities of visual perception among patients with retinal degeneration that may arise from hyperactivity. Next, I consider the disruption of neural encoding and anatomic connectivity that may result within the retina and in downstream visual centers of the brain. I then consider how specific characteristics of hyperactivity may distinguish various forms or stages of retinal degeneration, potentially helping in the near future to refine diagnosis and/or treatment choices for different patients. Finally, I review how consideration of these features may help optimize pharmacologic, gene, stem cell, prosthetic or other therapies to forestall visual loss or restore sight.

## Inner Retinal Function in Outer Retinal Disease

An ever-widening variety of genetic defects and biophysical mechanisms is recognized to cause progressive dysfunction and death of photoreceptors: hereditary retinal degenerations. Beyond this is an even broader array of neurologic and systemic disorders whose manifestations include retinal degeneration.

Gradual loss of vision in these patients is, of course, expected as the primary light-sensing cells of the retina fail, and so scientific attention in these diseases long has focused on photoreceptors. Yet beyond these cells the neural circuits of the inner retina perform a tremendous amount of visual processing before information about the world we see is “compressed” and sent to visual centers in the brain. Arguably, the retina itself may be considered a structure of the central nervous system (CNS), a perspective that may be helpful to understand its normal function and deterioration with disease.

## Potential Effects of Inner Retinal Hyperactivity on Visual Perception

Up to 70% of patients with retinal degenerations report an impressive variety of visual symptoms beyond a simple scotoma (“blacking out” of a region of visual space). These range from photopsias and scintillations to complex formed hallucinations to imagined scenes or awakened visual memories (Heckenlively et al., 1988; Murtha and Stasheff, 2003; Bittner et al., 2009, 2011; Brown et al., 2015), reflecting the varied ways in which the retina’s complex visual processing may be disrupted, not to mention the function of CNS visual pathways further downstream that form the conscious experience of the outside world, as such disease(s) progress (Ashtari et al., 2014).

The more complex of these abnormal visual perceptions may be considered a form of the Charles Bonnet syndrome originally described in 1760 in a patient with severe bilateral cataracts, but also reported for other forms of severe bilateral visual loss [e.g., age-related macular degeneration (AMD), diabetic retinopathy, or bilateral visual cortical injury] (Ffytche, 2005; Hedges, 2007; Cammaroto et al., 2008; Ffytche, 2009; Pang, 2016). In such cases, an analogy may be drawn to the “phantom limb” perception that may be caused by peripheral nervous system injury, and that appears to result at least partly from spontaneous hyperactivity of the injured neurons (Rachmachandran, 1992; Cammaroto et al., 2008; Schadlu et al., 2009; Pirowska et al., 2014; Luo and Anderson, 2016). Several articles in this Research Topic highlight the emergence of spontaneous hyperactivity among retinal ganglion cells (RGCs) in animal models of retinal degeneration, including the often oscillatory nature of such hyperactivity, and several different neural circuits in both inner and outer retina that can generate it (Barrett et al., 2015; Euler and Schubert, 2015; Ivanova et al., 2015b; Soto and Kerschensteiner, 2015; Trenholm and Awatramani, 2015; Goo et al., 2016; Tu et al., 2016; Zeck, 2016). Others have even drawn an analogy to epilepsy (Menzler and Zeck, 2011), although that disease category includes a plethora of individual diseases with widely variable manifestations and mechanisms (at least 30 broad but distinct types, according to the most recent international classification), most of which originate from CNS structures outside primary visual pathways (Fisher, 2017; Fisher et al., 2017).

How might this spontaneous hyperactivity among RGCs affect the vision of patients with retinal degeneration? It is easy to speculate that irregularly occurring bursts of such activity in RGCs, either individually or among groups of RGCs, would generate brief flashes of light, and that characteristics might vary substantially depending upon which functional type of RGC(s) were active at any given moment (Margolis and Detwiler, 2011; Menzler and Zeck, 2011; Yee et al., 2014). Alternatively, the excessive activity also may be viewed as “background noise” with the potential to interfere with the transmission of normal signals that create visual percepts when received and “decoded” by various visual center circuits in the brain. In its most simplistic conceptualization, the signal-to-noise ratio (SNR) may be decreased, simply in terms of the total rate of action potentials discharged by ganglion cells (Barrett et al., 2015; Ivanova et al., 2015b).

However, it now is widely accepted that a simple rate code cannot carry all the visual information conveyed by the retina to the brain (Baccus, 2007; Field and Chichilnisky, 2007; Schwartz and Berry, 2008; Jacobs et al., 2009; Koepsell et al., 2009; Sincich et al., 2009b; Tkacik et al., 2010; Xiao et al., 2013; Chaisanguanthum et al., 2014). A variety of more complex neural codes have been proposed to carry important visual information (Tkacik et al., 2010; Nirenberg and Pandarinath, 2012; Xiao et al., 2013; Chaisanguanthum et al., 2014; Marre et al., 2015; Rucci and Victor, 2015; Ioffe and Berry, 2017). These readily could incorporate mechanisms for complex and non-linear interactions between RGC spontaneous hyperactivity and “meaningful” activity that carries visual information. For example, vigorous light stimulation of normal retinas can provoke spontaneous RGC hyperactivity similar to that seen in retinal degenerations (Menzler et al., 2014). Conversely, electrical stimulation of degenerate retinas can reset the rhythm of spontaneous, oscillatory RGC hyperactivity (Ryu et al., 2010).

Classic Shannon information theory and variants of it have been employed to estimate the proportion of visual information that may be conveyed by some of these coding strategies (Shannon, 1948; Field and Chichilnisky, 2007; Sincich et al., 2009a; Wu and Srivastava, 2011). These theories also might be used to estimate the visual information lost due to hyperactivity disrupting the spatiotemporal structure of RGC spike trains in retinal degenerations. Alternatively, under certain conditions such as low contrast, visual noise may actually *enhance* visual perception: a principle known as stochastic resonance (Patel and Kosko, 2005; Kim et al., 2006; Funke et al., 2007; Trevino et al., 2016; van der Groen and Wenderoth, 2016). Too much, too little, or different types of noise, on the other hand, make images more difficult to perceive. In other animal models, RGC hyperactivity has been shown to disrupt the responses of *central* visual system neurons to light stimulation (Soto et al., 2012; Ivanova et al., 2015a; Soto and Kerschensteiner, 2015; Crair and Mason, 2016; Wang et al., 2016).

Just how such findings in animal models of retinal degeneration will translate to human visual perception in these diseases remains unclear. One framework for understanding how the central visual system may interpret or respond to abnormally elevated background RGC activity was developed initially to help understand amblyopia, and more recently also was applied successfully to patients with retinal degenerations: the concept of intrinsic equivalent noise (Pelli et al., 2004; McAnany et al., 2013). Patients are presented with standardized midrange contrast images to measure visual acuity with or without added background luminance “white noise.” The difference in a patient’s quantitative visual acuity measured in the presence vs. absence of this additional stimulus “noise” can be used to calculate the “efficiency” and “equivalent noise” of their visual perception. Thus, patients with various forms of hereditary retinal degeneration perform on such tests as if they perceive – beyond the extrinsic visual stimulus – additional background noise intrinsic to the central visual system, noise that control subjects do not perceive (McAnany et al., 2013).

Aside from informing our understanding of how RGC hyperactivity may directly affect the visual perception of patients with retinal degenerations, these relatively simple methods might also prove useful as tools for clinical screening of patients with retinal degenerations, perhaps to detect more subtle deterioration of vision at earlier stages of disease progression. In turn, this might enable earlier, more effective treatments, and/or identify optimal treatment candidates.

The precise nature of RGC hyperactivity may vary with the specific form of retinal degeneration. For example, Goo et al. (2011a,b, 2016) have demonstrated subtle differences in the dominant frequency of oscillation in spontaneous activity between the *rd1* and *rd10* mouse models of retinitis pigmentosa, and at different developmental stages of *rd10* degeneration. Our own studies have demonstrated differences in the power spectrum density distribution of this activity between mouse models of two common forms of Leber’s congenital amaurosis (LCA), *Cep290* and *Rpe65* (Stasheff et al., 2014). In some animal models, specific intra-retinal synaptic pathways are preferentially altered in the face of others that remain unchanged (Soto and Kerschensteiner, 2015; Tu et al., 2016).

It also has been long recognized that changes in the frequency composition of RGC activity – particularly during select periods of early visual system development – can substantially alter the distribution of RGC axon projections to central visual system targets such as the lateral geniculate nucleus, superior colliculus, and visual cortex (Hanganu et al., 2006; Rebsam et al., 2009; Stafford et al., 2009; Ackman et al., 2012; Burbridge et al., 2014). Thus, variations in specific parameters of RGC hyperactivity among different forms of retinal degeneration may translate to differences in the visual perception of patients with these varied diseases, either directly or by the way they impact downstream processing of visual information in central visual pathways (lateral geniculate, primary, extrastriate, and associational cortices). It should be noted that although RGC spontaneous hyperactivity has been demonstrated consistently by many researchers and in multiple animal models, and abnormal visual percepts described by many patients across a broad variety of retinal diseases, a direct causal relationship between the two remains essentially speculative. Sufficiently sensitive technologic methods are not yet available to detect human RGC hyperactivity reliably or, conversely, to assess conscious visual perceptions of laboratory animals with retinal degeneration.

## Implications for Diagnosis and Treatment of Retinal Degenerations

How can further understanding of the spontaneous RGC hyperactivity that accompanies retinal degenerations contribute to improved clinical diagnosis and treatment of these diseases? I have already discussed how the incorporation of specialized but simple psychophysical testing such as low contrast acuity with or without added “noise” (Pelli et al., 2004; McAnany et al., 2013) may improve diagnostic sensitivity, so that these diseases may be detected earlier. Perhaps particular forms of retinal degeneration also may be distinguished at an earlier stage of disease, enabling earlier and more targeted treatment. If proven sufficiently sensitive and specific in the future, such testing might be useful for staging disease, selecting optimal patients for treatment, and/or predicting treatment responsiveness.

To the extent that RGC hyperactivity plays a critical role in establishing retinal and central circuits for visual processing during an early developmental period, its emergence as retinal degenerations progress may help explain the considerably greater effectiveness of treatments such as gene therapy in young children vs. adults (Maguire et al., 2009; Ashtari et al., 2015). Direct modulation of RGC hyperactivity – perhaps as a primary therapy, or as a complementary adjunctive treatment – might improve outcomes and/or delay disease (Toychiev et al., 2013; Barrett et al., 2015, 2016). Cutting-edge treatments such as electrical stimulation via a visual prosthesis (Weiland et al., 2016; Cheng et al., 2017; Mills et al., 2017) may allow very specific modulation of RGC activity so as to counteract excessive “random noise,” and/or fine tune the complex signals that encode visual information sent to the brain (Sekirnjak et al., 2008; Freeman et al., 2011; Nirenberg and Pandarinath, 2012; Jepson et al., 2014; Im and Fried, 2015). Finally, to the extent that RGC hyperactivity can be attributed to specific neurotransmitter systems in particular forms of retinal degeneration (Biswas et al., 2014; Liu et al., 2015; Ivanova et al., 2016; Tu et al., 2016), it may be possible to selectively modulate the responsible systems and improve the effective “signal-to-noise ratio” of retinal signaling, and thus improve overall conscious visual perception in these patients.

## Summary and Conclusion

Over the past decade, there has been an acceleration of discoveries revealing that hereditary retinal degenerations involve not only abnormal function of the outer retina (photoreceptors and support cells) but also substantial reorganization of circuitry and electrophysiologic functioning of the inner retina, culminating in abnormal output of RGCs. This includes an increase in background spontaneous activity originating from multiple sites, from the level of photoreceptors, horizontal cells, and bipolar cells to the output level of RGCs. The aberrant activity often is rhythmic and oscillatory and may affect changes in neural connectivity and function in further downstream visual centers of the CNS.

The discovered background spontaneous hyperactivity of RGCs may help explain the variety of phosphenes and other visual phenomena experienced by many patients in some stages of retinal degeneration. It may also degrade normal visual perception, but alternatively might actually improve perception under certain conditions (e.g., via stochastic resonance). Individual visual processing pathways may be selectively affected, causing specific distortions in visual perception, and even translate to complex visual phenomena – including formed hallucinations – at a cortical level. Because of disruptions in normal developmental maturation of these retinal circuits, early identification and treatment of these disorders may be critical for developing therapies that ultimately are more effective. Finally, basic mechanisms identified to date do suggest some further avenues to explore in order to improve clinical testing of patients, for both diagnosis and the identification of optimal candidates for specific treatments in clinical trials.

There is a great deal yet to be learned about the clinical implications of these findings for patients with retinal degeneration. However, studies of animal models such as those in this Research Topic highlight a variety of key features of this reorganization and suggest previously unrecognized mechanisms that may explain patients’ symptoms. Because certain of these characteristics appear to be disease-specific, they also may help to diagnose specific disorders earlier and more accurately. For example, current clinical tests such as low-contrast visual acuity charts may be adapted to assess for intrinsic “equivalent noise” and potentially quantify disease stage, perhaps even predict treatment responses in individual patients. Ultimately, deeper understanding of the inner retina’s reorganization may pave the road to more mechanistically targeted therapies and provide more specific tests to screen for optimal subjects in clinical trials of new and evolving treatments.

## Author Contributions

SS reviewed relevant literature, wrote and revised the manuscript, read and approved the submitted version.

## Conflict of Interest Statement

The author declares that the research was conducted in the absence of any commercial or financial relationships that could be construed as a potential conflict of interest.
